# rTMS-Induced Changes in Glutamatergic and Dopaminergic Systems: Relevance to Cocaine and Methamphetamine Use Disorders

**DOI:** 10.3389/fnins.2020.00137

**Published:** 2020-03-06

**Authors:** Jessica Moretti, Eugenia Z. Poh, Jennifer Rodger

**Affiliations:** ^1^Experimental and Regenerative Neurosciences, School of Biological Sciences, The University of Western Australia, Crawley, WA, Australia; ^2^School of Human Sciences, The University of Western Australia, Crawley, WA, Australia; ^3^Brain Plasticity Group, Perron Institute for Neurological and Translational Science, Nedlands, WA, Australia

**Keywords:** rTMS, addiction, brain stimulation, cocaine use disorder, methamphetamine use disorder, glutamatergic system, dopaminergic system

## Abstract

Cocaine use disorder and methamphetamine use disorder are chronic, relapsing disorders with no US Food and Drug Administration-approved interventions. Repetitive transcranial magnetic stimulation (rTMS) is a non-invasive brain stimulation tool that has been increasingly investigated as a possible therapeutic intervention for substance use disorders. rTMS may have the ability to induce beneficial neuroplasticity in abnormal circuits and networks in individuals with addiction. The aim of this review is to highlight the rationale and potential for rTMS to treat cocaine and methamphetamine dependence: we synthesize the outcomes of studies in healthy humans and animal models to identify and understand the neurobiological mechanisms of rTMS that seem most involved in addiction, focusing on the dopaminergic and glutamatergic systems. rTMS-induced changes to neurotransmitter systems include alterations to striatal dopamine release and metabolite levels, as well as to glutamate transporter and receptor expression, which may be relevant for ameliorating the aberrant plasticity observed in individuals with substance use disorders. We also discuss the clinical studies that have used rTMS in humans with cocaine and methamphetamine use disorders. Many such studies suggest changes in network connectivity following acute rTMS, which may underpin reduced craving following chronic rTMS. We suggest several possible future directions for research relating to the therapeutic potential of rTMS in addiction that would help fill current gaps in the literature. Such research would apply rTMS to animal models of addiction, developing a translational pipeline that would guide evidence-based rTMS treatment of cocaine and methamphetamine use disorder.

## Introduction

Substance dependence is a chronic, relapsing disorder with significant monetary and societal costs. Moreover, there are still substance use disorders with no US Food and Drug Administration (FDA)-approved interventions, such as cocaine use disorder and methamphetamine use disorder. Therefore, there is a need to investigate possible treatments and interventions that could help combat these addictions. One avenue of investigation is the use of non-invasive brain stimulation techniques, such as repetitive transcranial magnetic stimulation (rTMS). rTMS therapy has been FDA approved for treatment-resistant depression (O’Reardon et al., 2007; Horvath et al., 2010) and obsessive-compulsive disorder (Carmi et al., 2018) and has also shown promise in several other neurological disorders where its ability to induce plasticity proves useful (Fregni and Pascual-Leone, 2007; Pell et al., 2011; Lefaucheur et al., 2014). The aim of this review is to highlight what is currently known about the effects of rTMS within the field of addiction, specifically on cocaine and methamphetamine dependence. In this review, we consider human and animal studies, which together allow us to relate the outcomes of rTMS therapy to the neurobiological mechanisms that seem most involved in addiction – changes in the glutamatergic and dopaminergic systems.

## Major Pathways Involved in Addiction

Addiction is a complex condition that involves several neural pathways and mechanisms of dependence that can be specific to the substance of abuse. Broadly speaking, however, the main pathways implicated in addiction are the glutamatergic afferents from the prefrontal cortex (PFC) to the nucleus accumbens (NAc) of the ventral striatum and ventral tegmental area (VTA) of the midbrain, and the dopaminergic efferents from the VTA to the striatum. Abnormal function of these pathways in addiction results in the disruption and dysregulation of dopaminergic activity (Koob and Volkow, 2010). Together these pathways are referred to as the mesocorticolimbic system.

Drug addiction is characterized by changes at all points of the mesocorticolimbic system. Exposure to addictive substances such as cocaine and methamphetamine is accompanied by a fast and steep release of dopamine in the NAc (Volkow et al., 2007; Fowler et al., 2008), affecting mesocorticolimbic pathways and characterizing the first stage of addiction – intoxication (Koob and Volkow, 2010). Although transient, this substance-induced elevation in dopamine may exceed that observed following “normal” physiological processes (Volkow et al., 2007). Several other neurotransmitters, including opioid peptides (Daunais et al., 1993; Spangler et al., 1993), serotonin (see Müller and Homberg, 2015), and acetylcholine (Imperato et al., 1993; Zocchi and Pert, 1994; Berlanga et al., 2003), are also increased during the intoxication stage (Koob and Volkow, 2010).

Repeated exposure to addictive substances can result in maladaptive sensitization within the mesocorticolimbic system, specifically toward dopamine release, whereby conditioned incentive sensitization (increase in “wanting” without necessarily a change in “liking”) toward drug-associated stimuli occurs (Berridge and Robinson, 2016). The PFC to NAc glutamatergic pathway, which includes afferents from the dorsolateral PFC (DLPFC), is involved in modulating these value signals (Hayashi et al., 2013). Chronic drug use may also induce long-term neuroadaptations as a result of the repeated hyperactivity of dopaminergic transmission, for example, facilitating the development of learned associations between drug-related cues, such as images or videos of drugs, drug paraphernalia in an experimental setting, the anticipation of drug intoxication, and the accompanying physiological changes such as the induction of dopamine release in the striatum (Wolf et al., 2004; Berridge and Robinson, 2016). Such neuroadaptations may underpin the impact of cues, which are usually specific to the drug of interest and induce an increase in striatal dopamine that is thought to underlie craving (Goldstein and Volkow, 2002; Volkow et al., 2006, 2008; Volkow and Morales, 2015). Neuroplastic changes from chronic drug use are also associated with reduced cognitive control, compulsive drug use, and impulsivity to continue addictive behavior (Koob and Volkow, 2010).

It is also thought that the inability to inhibit drug-seeking behaviors is partly underpinned by a weakened executive control network and PFC dysfunction (Bechara, 2005; Hu et al., 2015; Ekhtiari et al., 2019), which are thought to contribute to the development of behaviors that are characteristic of addiction (Volkow and Fowler, 2000; Goldstein and Volkow, 2002). The PFC is made up of several regions that may each contribute to different aspects of addictive behavior (for a review, see Goldstein and Volkow, 2011). For example, the ventrolateral PFC and lateral orbitofrontal cortex are linked with habitual responding and therefore linked with impulsivity and inflexible behavior patterns. In contrast, the ventromedial PFC, which includes the subgenual anterior cingulate cortex (ACC) and medial orbitofrontal cortex, is linked with emotion regulation and incentive salience of drugs and related cues (Goldstein and Volkow, 2011). Furthermore, the DLPFC has a significant role in top-down control and metacognitive functions such as attention bias, motivation, and self-control, among others (Goldstein and Volkow, 2011). It is therefore important to be mindful when reading the literature that different PFC regions can be associated with particular cognitive processes and can also have different anatomical connections and feedback loops.

Contributing to the addiction cycle are the acute withdrawal effects, which include reduced reward sensitivity and motivation for natural rewards (Barr and Phillips, 1999). Cessation of drug use is associated with altered levels of a number of different substances, including a decrease in basal dopamine levels in the striatum (e.g., Rossetti et al., 1992; Weiss et al., 1992). Evidence of the hypodopaminergic tone observed within the mesolimbic system from both experimental and clinical studies led to the development of the dopamine hypothesis of drug addiction (Melis et al., 2005), and progress within the field has been reviewed more recently (Fattore and Diana, 2016). Hypodopaminergic tone has also been associated with a decrease in striatal dopamine terminal density (Lee et al., 2011) and downregulation of dopamine D_2_ receptors expressed on both presynaptic and postsynaptic neurons, the latter being important for inhibitory feedback signals (Nutt et al., 2015; Volkow and Morales, 2015). These have been linked to pathological behaviors such as impulsivity and compulsive drug seeking in subjects addicted to methamphetamine and cocaine (Lee et al., 2009; Moeller et al., 2018). Changes within the dopaminergic system contribute to the acute withdrawal effects, which include reduced reward sensitivity and motivation for natural rewards (Barr and Phillips, 1999), as well as negative affect, such as irritability, states of stress, and malaise (Baker et al., 2006; Fox et al., 2008; Koob, 2009; Koob and Volkow, 2010). This negative state of withdrawal tends to further narrow behavior toward drugs and drug-related stimuli, perpetuating drug use.

## Manipulating Circuits Involved in Addiction

Our current knowledge of the circuits involved in addiction comes from animal studies as the pathways and brain regions involved are similar in rodents and humans (Kalivas et al., 2006; Madeo and Bonci, 2019). Animal models of addiction are one of the most well-developed and validated models in neuropsychiatric research and are used by researchers and clinicians to gain insight into some of the mechanisms involved in addiction (Kalivas et al., 2006; Venniro et al., 2016). These findings have since been supported by follow-up studies that alter activity in a targeted brain region (Conrad et al., 2008; Chen et al., 2013; Venniro et al., 2016). This has been done mostly in one of two ways: direct electrical stimulation and, more recently, optogenetics.

Direct evidence of brain stimulation altering compulsive drug-seeking behaviors has been shown following application of localized electrical stimulation to the PFC of cocaine-addicted rats and mice *via* implanted electrodes (Levy et al., 2007). Following 20-Hz stimulation (30 min, 10 pulses/train, one train every 2 s) in the PFC, cue-induced cocaine-seeking behavior and motivation for its consumption were reduced (Levy et al., 2007), which is likely related to the release of dopamine and glutamate in the NAc following stimulation in the PFC (Taber et al., 1995; You et al., 1998). Comparison of various stimulation frequencies in the medial PFC (mPFC) showed that 10- to 20-Hz electrical stimulation that lasted >5 s resulted in peak extracellular dopamine levels, compared to 30-, 40-, and 60-Hz stimulation frequencies, possibly due to its similarities to endogenous bursting rhythms of the VTA (Hill et al., 2018).

Since the development of genetic techniques such as optogenetics (Boyden et al., 2005; Han, 2012), researchers have been able to manipulate neural circuits with greater specificity (e.g., purely glutamatergic neurons) to gain a better understanding of the circuits involved in pathological drug-seeking behavior. It is important to note, however, that caution must be taken when interpreting results of studies that utilize optogenetics methods, and inclusion of rigorous control groups is necessary (see Tye and Deisseroth, 2012). For example, certain illumination protocols can induce temperature fluctuations within the surrounding tissue, affecting behavioral outcomes (Owen et al., 2019). Therefore, control experiments should include a viral construct that does not encode for light-sensitive ion channels (Yizhar et al., 2011; Owen et al., 2019). Despite these limitations, a study has shown that optogenetics stimulation of hypoactive glutamatergic neurons of the PFC can modulate compulsive drug seeking in cocaine-addicted rats (Chen et al., 2013). Using adeno-associated viruses, light-sensitive ion channels [channelrhodopsin for depolarization and halorhodopsin for hyperpolarization (Tye and Deisseroth, 2012)] were transfected into glutamatergic neurons of the prelimbic cortical area. Activation of the transfected neurons (1 Hz, 10-ms wide pulses, 10–15 mW, 473 nm) *via* channelrhodopsin led to reduced compulsive drug-seeking behavior, whereas inhibition with halorhodopsin led to increased drug-seeking behavior (Chen et al., 2013). Therefore, it appears that excitatory stimulation of PFC glutamatergic efferents can rescue its hypoactivity and may result in downstream effects that can increase dopamine transmission, ultimately reducing compulsive drug seeking in addicted subjects.

The dynamic plasticity of the mesocorticolimbic pathways is thus central in addiction, particularly the maladaptive changes that occur within glutamatergic and dopaminergic systems, and offers a compelling target for therapeutic interventions to modulate circuit activity. In order to translate these findings into humans and manipulate the activity of relevant circuits for therapeutic purposes, many studies have used rTMS, which allows non-invasive modulation of brain activity. Studies with rTMS can vary in which stage of the addiction cycle they lie; however, most clinical studies on cocaine and methamphetamine addiction tend to focus on patients who are in the preoccupation/anticipation stage after chronic withdrawal from the drug. Therefore, this review will focus on the anticraving effects of rTMS on substance dependence, with a particular focus on cocaine and methamphetamine dependence. The aim of this review is to highlight potential neurobiological mechanisms that can guide future rTMS research within the field.

## Fundamentals of *r*TMS

Repetitive TMS has shown promising results for the treatment of a range of neurological disorders and has been shown to induce plasticity in humans, as measured *via* changes in corticospinal excitability (Pell et al., 2011) and alterations in mood, behavior, and cognition (e.g., O’Reardon et al., 2007; Luber and Lisanby, 2014). Currently FDA approved for major depressive disorder and obsessive-compulsive disorder, this non-invasive brain stimulation technique may also facilitate recovery from substance use disorders. Reasons for how rTMS induces therapeutic effects in various neurological disorders remain unclear; however, a number of preclinical studies have identified mechanisms that could underlie the long-term effects. These mechanisms include alterations to neuron excitability (Sun et al., 2011; Hoppenrath et al., 2016; Tang et al., 2016) and Hebbian-type strengthening of synapses (Vlachos et al., 2012; Lenz et al., 2015), as well as alterations to gene expression (Ikeda et al., 2005; Grehl et al., 2015), trophic factors necessary for neuroplasticity (Gersner et al., 2011; Rodger et al., 2012; Makowiecki et al., 2014), activity within brain regions beyond the induced electrical field (Aydin-Abidin et al., 2008; Seewoo et al., 2018, 2019), and even changes to non-neuronal cells, which may contribute to plastic events (Clarke et al., 2017a, b; Cullen et al., 2019).

Utilizing the principles of Faraday’s law of electromagnetic induction, rTMS is delivered via a coil positioned above the scalp to induce electrical currents in the underlying brain tissue. These electrical currents have the capacity to induce neuroplasticity, either by triggering action potentials in the underlying cortical neurons (Pashut et al., 2014; Li et al., 2017), or by modulating neuronal excitability (Sun et al., 2011; Hoppenrath et al., 2016; Tang et al., 2016). Effects of rTMS depend on multiple stimulation parameters, such as the frequency and rhythm of the pulses delivered, number of pulses, coil and pulse shape, stimulation intensity, and number of sessions (Pell et al., 2011; Rodger and Sherrard, 2015). In addition, morphological differences such as the brain tissue shape (e.g., gyral anatomy) relative to the device can influence rTMS effects (Wagner et al., 2009; Thielscher et al., 2011).

### Frequency and Pulse Number

In humans, alteration to corticospinal excitability is the main measure of rTMS-induced plasticity. Changes in excitability can be measured by comparing motor-evoked potentials (MEPs) before and after stimulation. MEPs are recorded by applying a single TMS pulse at a specified intensity to the motor cortex and recording the electromyogram of a peripheral muscle. Changes to human cortical excitability have been shown to be frequency dependent, with a simple high-frequency (HF) (≥5 Hz) or low-frequency (LF) (<1 Hz) rTMS protocol able to increase or decrease excitability, respectively (Hallett, 2007; Pell et al., 2011), albeit with high intraindividual and interindividual variability (Ridding and Ziemann, 2010; Hinder et al., 2014; Hamada and Rothwell, 2016). There are also complex patterned protocols, such as theta burst stimulation (TBS), which utilize a train consisting of three pulses at 50 Hz, repeated at 5 Hz, for a total of 600 pulses (although other variants also exist). TBS protocols can be differentiated into two subtypes: continuous (cTBS), wherein 20 trains of uninterrupted pulses are delivered, and intermittent (iTBS), with a 2-s TBS train repeated every 10 s. Intermittent TBS has been shown to have excitatory effects on cortical excitability, whereas cTBS has inhibitory effects (Huang et al., 2005). Compared to simple protocols, these complex patterned protocols may be more effective for inducing long-term changes, with an increase in MEPs induced by iTBS lasting for approximately 60 min (Wischnewski and Schutter, 2015). Recently, an analysis of various rTMS protocols has suggested that frequency is the strongest predictor of the direction of change in cortical excitability, as measured via MEPs (Wilson and St George, 2016).

An additional contributor to frequency effects is the pulse rhythm, or the pattern in which trains of frequency are delivered. There is a wide variety of pulse numbers and pulse rhythms used in the literature, and it is not clear what effect these factors have on rTMS efficacy, and if there is a dose dependency. Train length and intertrain intervals are determined in part by the characteristics of the rTMS device: every pulse generates heat in the coil, and more heat is generated at higher frequencies (Weyh et al., 2005). It is therefore necessary to introduce intertrain intervals to allow the coil to cool down. Human studies suggest that pulse number and train number are not related to the outcome of rTMS in a straightforward way (Huang et al., 2005; Hamada et al., 2013), but results are difficult to interpret because of variability in human subjects. One study specifically explored the effect of pulse number on expression of protein markers in the cortex of healthy rats (Volz et al., 2013). For TBS protocols, increasing the number of pulses did not lead to a simple dose-dependent change, but rather elicited a “waxing-and-waning” effect for the markers of inhibitory interneuron and γ-aminobutyric acid (GABA) activity (Volz et al., 2013). Furthermore, increasing number of pulses led to a progressive reduction in protein expression of the immediate early gene c-Fos, which normally reflects neuronal activation (see Aydin-Abidin et al., 2008). Surprisingly, the reduction occurred following both inhibitory (cTBS) and excitatory (iTBS) protocols (Volz et al., 2013), suggesting a complex relationship between the number and rhythm of pulses and the effect on cortical neurons.

### Intensity

The strength of stimulation is a variable parameter. In order to account for interindividual changes in excitatory thresholds, the intensity of rTMS is often applied as a percentage of the resting motor threshold (rMT). Techniques to find a participant’s rMT vary, but it is defined as the lowest stimulation intensity that produces at least five MEPs (≥50 μV) out of 10 consecutive stimuli (Rossini et al., 1994). Intensity will usually be set at a % between 80 and 120% rMT, depending on the study. For TBS, lower intensities of 80–90% are usually used, which contribute to its improved tolerability (Oberman et al., 2011). Higher intensities are often associated with more adverse effects (Rossi et al., 2009) but are more likely to elicit action potentials (≥100% rMT), which could have stronger cortical effects. Nonetheless, stimulation below motor threshold (80–95% rMT) is still capable of eliciting cortical and subcortical changes in distinct networks across the brain (Bestmann et al., 2004).

Experimental animal models have shown that high-intensity rTMS [≥1 Tesla (T)] can evoke action potential firing (Pashut et al., 2014; Li et al., 2017) and alter neurotransmitter concentrations (e.g., Ben-Shachar et al., 1997), whereas low-intensity rTMS (≤120 mT) can lower action potential thresholds and increase spike firing frequency for up to 20 min after magnetic stimulation (Tang et al., 2016). In addition, behavioral changes in a mouse model of depression have been shown to be dependent on stimulation intensities (Heath et al., 2018). Low-intensity effects may also contribute to the impact of high-intensity protocols in humans due to the wide distribution of low-intensity magnetic fields within brain tissue outside the site of focal stimulation (Bestmann et al., 2004). Within the field of magnetic stimulation, a limitation is the inconsistency of reporting the induced field intensities (see, for example, Table 1, which reports the intensity listed in the original research articles). Some articles mention the induced magnetic field, the induced electric field or a % output of the machine required to evoke an observable muscle twitch (MEP). Adding to this confusion, different units of measurement have also been reported (e.g., mT, V/m, and dB/dT).

**TABLE 1:** rTMS effects on dopaminergic systems sorted by sampling method used.

**Study**	**Subject**	**Session number**	**rTMS parameters**	**rTMS coil^a^ and target**	**Sampling method**	**Sampling time**	**Significant effect**
Zangen and Hyodo, 2002	Rat	Single	2 Hz, 100 s, 500 V/s	5.4-cm circular coil. Over the head, rostral, or caudal side	Microdialysis: DA, DOPAC, HVA	During, 0–45 min pms, 15-min intervals	*NAc*: ↑ DA after rostral or caudal stimulation, returned to baseline within 15 min pms
Keck et al., 2000	Rat	Single	20 Hz, 2.5 s, 2 min ITI, 20 trains, Σ1,000 pulses, 130% MT	5.7-cm circular coil, left FC	Microdialysis: DA, DOPAC, HVA	Baseline, 0–60 (rTMS), 90–120 min pms, 30-min intervals	*Urethane anesthetized – right hippocampus*: ↑ DA 60 and 90 min pms
Keck et al., 2002	Rat	Single	20 Hz, 2.5 s, 2 min ITI, 20 (i) or 6 (ii) trains, Σ1000 or Σ600 pulses, 130% MT	5.7-cm circular coil, left FC	Microdialysis: DA, DOPAC, HVA	(i) Baseline, 0–60 (rTMS), 90–180 min pms, 30-min intervals. (ii) Baseline, 0–30 (rTMS), 60–180 min pms, 30-min intervals	*(i) Urethane anesthetized – right hippocampus*: same as Keck et al. (2000); *right NAc shell*: ↑ DA 120*–*180 min pms; *right dorsal striatum*: ↑ DA 90–180 min pms. *(ii) Awake – right hippocampus*: ↑ DA 90–180 min pms; *right NAc shell*: ↑ DA 30–180 min pms
Erhardt et al., 2004	Rat	Single	20 Hz, 2.5 s, 2.5 min ITI, six trains, Σ300 pulses, 130% MT	5.7-cm circular coil, left FC	Microdialysis: DA	Baseline, 0–30 (rTMS), 60–120 min, 30-min intervals	*Right NAc shell*: ↑ DA at 0–30 min for morphine sensitized rats + rTMS vs. basal, saline + rTMS, morphine + sham; ↑ DA at 60, 90 morphine + rTMS vs. basal, morphine + sham; ↑ DA at 120 min vs. sham + morphine
Kanno et al., 2004	Rat	Single	25 Hz, 1 s, 1 min ITI, 20 trains, Σ500 pulses, 0.2 T, 0.6 T, and 0.8 T	7-cm F-o8 coil, FC	Microdialysis: DA	Baseline, 0–20 (rTMS), 40–180 min, 20-min intervals	*0.6 T:*↑ DA in dorsolateral striatum for 0–130 min, ↑ DA 0–50 min in PFC; 0.2 and 0.8T: no change
Poh et al., 2019	Mouse	Single	10 Hz, one train, Σ3,600 pulses, 1.2 T	7.5-cm F-o8 coil, over the head	Homogenates: DA, DOPAC, HVA	Immediately after last session	*Striatum*: ↑ DOPAC
Ben-Shachar et al., 1997	Rat	Single	25 Hz, 2 s, one train, Σ50 pulses, 2.3 T	5-cm coil, over the head	Homogenates: DA, DOPAC, HVA	5 s after last session	*FC*: ↓ DA, ↑ HVA, ↑ turnover; *hippocampus*: ↑ DA, ↓ turnover; *striatum*: ↑ DA, ↑ DOPAC; ↓ turnover; *midbrain*: ↓ HVA
Strafella et al., 2001	Human	Single	Three blocks separated by 10 min: 10 Hz, 1 s, 10 s ITI, 15 trains, Σ450 pulses, 100% rMT^∗^	9-cm circular coil, left DLPFC	PET study: [^11^C] raclopride BP	Within 65 min pms	*Ipsilateral caudate*: ↓ DA binding potential, suggesting ↑ DA release
Ko et al., 2008	Human	Single	cTBS, 20 s, three trains, Σ900 pulses, 80% AMT	F-o8 coil, left and right DLPFC.	PET study: [^11^C] raclopride BP	Within 60 min pms	*Left DLPFC – ipsilateral caudate-putamen and contralateral caudate nucleus*: ↓ DA binding potential, suggesting increase DA release. *Right DLPFC:* no change in regions examined
Cho and Strafella, 2009	Human	Single	Three blocks separated by 10 min: 10 Hz, 1 s, 10 s ITI, 15 trains, Σ450 pulses, 100% rMT^∗^	7-cm F-o8 coil, left and right DLPFC	PET study: [^11^C] raclopride BP	Within 95 min pms	*Left DLPFC – ipsilateral subgenual ACC, pregenual ACC, OFC:*↓ DA binding potential, suggesting increased DA release Right DLPFC: no change in regions examined
Strafella et al., 2003	Human	Single	Three blocks separated by 10 min: 10 Hz, 1 s, 10 s ITI, 15 trains, Σ450 pulses, 90% rMT^∗^	9-cm circular coil, left M1 or occipital cortex	PET study: [^11^C] raclopride BP	Within 65 min pms	*M1 – ipsilateral putamen:*↓ DA binding potential, suggesting increase DA release, when compared to ipsilateral OCC stimulation
Ohnishi et al., 2004	Macaque	Single	5 Hz, 20 s, 40 s ITI, 20 trains, Σ2,000 pulses, 35% max stimulator output	6.2-cm double-cone coil, right M1 cortex	PET study; [^11^C] raclopride BP	Within 60 min pms	*Anesthetized – bilateral ventral striatum (incl. NAc)*: ↓ DA binding potential, suggesting ↑ DA release; *ipsilateral putamen*: ↑ DA binding, suggesting decrease DA release. *Dorsal striatum*: no change
Pogarell et al., 2006	Human-depressed subjects	15 sessions	First session: 10 Hz, 10 s, 30 s ITI, 30 trains, Σ3,000 pulses, 100% rMT; followed by Σ 1,500 pulses	7-cm F-o8 coil, left DLPFC	SPECT study: [^123^I] IBZM BP	Before and 30 min after first session, before and after 15th session	*Bilateral striatum*: ↓ DA binding potential compared to pre-rTMS within each session, suggesting immediate ↑ DA release.
Pogarell et al., 2007	Human-depressed subjects	15 sessions	10 Hz, 10 s, 30 s ITI, 30 trains, Σ3,000 pulses, 100% rMT	7-cm F-o8 coil, left DLPFC	SPECT study: [^123^I] IBZM BP	Before and 30 min after first session, before and after 15th session	*Bilateral striatum*: ↓ DA binding potential compared to pre-rTMS within each session, suggesting immediate ↑ DA release. Similar results observed following exposure to D-amphetamine
Hausmann et al., 2002	Rat	Single or 14 sessions	20 Hz, 10 s, two trains, 400 pulses, 1 T	2.3-cm F-o8 coil, over the head	*In situ* hybridization, immunohistochemistry	12 h pms	*Ventral midbrain*: no difference in TH-mRNA or TH protein in all groups
Ikeda et al., 2005	Mouse	Single or 20 sessions	20 Hz, 2 s, 1 min ITI, 20 trains, 800 pulses, 0.75 T	7.5-cm round coil, over the head	RT-PCR: DAT mRNA, monoamine uptake, and ligand binding assay	1, 4, 12, 24 h pms (single and chronic) or 10 d pms (chronic)	*Single-cerebrum:*↑ DAT mRNA 4 and 24 h pms, ↓ DAT mRNA 12 h pms *Chronic-cerebrum*: ↑ DAT mRNA following 24 h and 10 d pms; *synaptosomes*: ↑ DA uptake, transport rate 24 h pms, no changes to affinity
Etiévant et al., 2015	Mouse	Single or five sessions	15 Hz, 10 s, 0.5 s ITI, three trains, 450 pulses, 53% MSO	5-cm Fo8	Western blot	Immediately after single session, 2 h, 5, 10, 20, 60 d pms (chronic)	*Single-PFC:* no change in D_2_R expression *Chronic-PFC:*↑ D_2_R expression 5 d pms

*^*a*^Outer diameter of each loop. ACC, anterior cingulate cortex; AMT, active motor threshold; BP, binding potential; cTBS, continuous theta burst stimulation; DA, dopamine; DAT, dopamine transporters; DLPFC, dorsolateral prefrontal cortex; DOPAC, 3,4-dihydroxyphenylacetic acid; FC, frontal cortex; F-o8, figure-of-eight; HVA, homovanillic acid; IBZM, iodobenzamide; ITI, intertrain interval; M1, primary motor cortex; MSO, maximum stimulator output; MT, motor threshold; NAc, nucleus accumbens; OFC, orbitofrontal cortex; PET, positron emission tomography; PFC, prefrontal cortex; pms, post magnetic stimulation; rMT, resting motor threshold; SPECT, single-photon emission computed tomography; TH, tyrosine hydroxylase.*

### Coil Parameters

There are several different coil designs available for rTMS, with changes to coil shape affecting the induced electric field in the brain. The coil properties of various designs have been characterized by Deng et al. (2013). Traditionally, coils can be separated into circular coils or figure-of-eight (F-o8) coils. Circular coils induce the greatest current intensity beneath the coil windings, whereas F-o8 coils have a focalized hotspot in the center of the coil where the windings of the two circular coils are the nearest to each other, with less intense peaks on the opposing outer rings (Deng et al., 2013). Because of this, F-o8 coils are usually used for their high focality.

The depth of stimulation of conventional circular and F-o8 coils, according to the definitions in Deng et al. (2013), ranges from 1.0 to 1.9 cm, and these coils are therefore limited to cortical stimulation. However, because many key structures lie below the cortex, there has been development of different coils to stimulate deeper structures, dubbed deep TMS (dTMS). The most popular coil design for dTMS is the H-coil (Zangen et al., 2005; Roth et al., 2007), of which there are now more than 20 different versions (Roth et al., 2013). H-coils are helmet-like and stimulate the brain bilaterally with a depth of up to 2.4 cm (Deng et al., 2013). However, to achieve this depth, the intensity of the induced stimulation is more diffuse than an F-o8 coil, stimulating a larger surface area with a relatively weaker electric field (Deng et al., 2013).

## *R*TMS in Cocaine and Methamphetamine Abuse – Clinical Research

**FIGURE 1:**
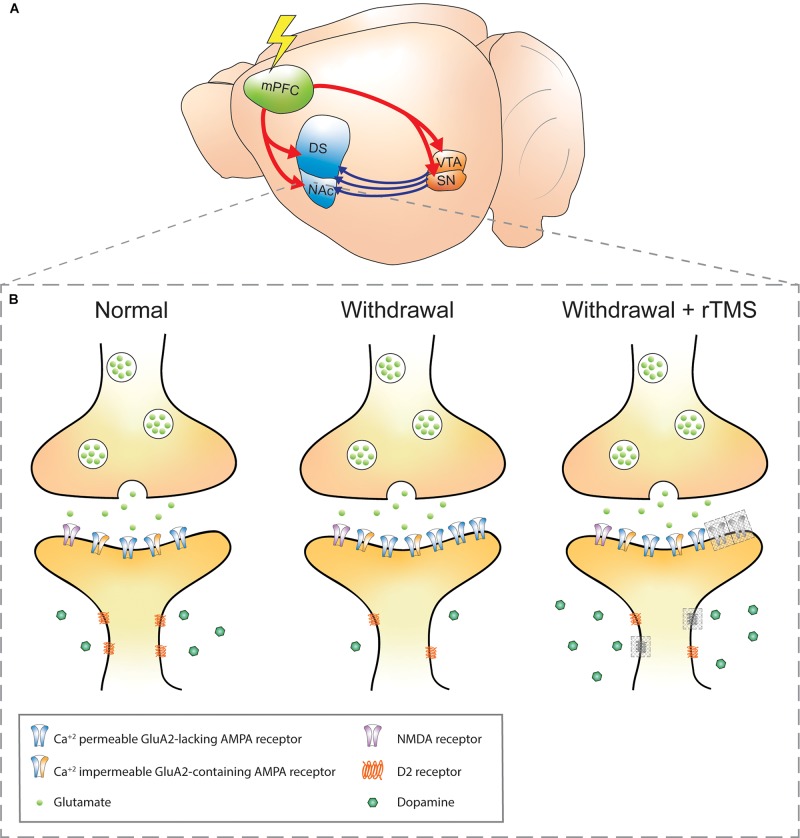
Schematic of addiction circuitry and the synaptic changes between an efferent mPFC glutamatergic neuron axon terminal and accumbal D_2_ receptors expressing MSN dendrite. **(A)** Rodent brain with glutamatergic efferents (red) projecting to the striatum and ventral midbrain nuclei. Dopaminergic projections (blue) from the VTA and SN project to the striatum. The rodent mPFC is comparable to the DLPFC in humans, a common site of rTMS stimulation in addiction (Diana et al., 2017). **(B)** Axon terminal of a mPFC glutamatergic neuron synapsing onto a D_2_ receptors-expressing MSN in the NAc in normal, withdrawal, and withdrawal + rTMS (proposed) treatment brain state. During cocaine or methamphetamine withdrawal, Ca^2+^-permeable GluA2-lacking AMPA receptors are upregulated in the NAc, which increases the sensitivity of NAc neurons to excitatory inputs and is a requirement for cue-induced drug craving (Cornish and Kalivas, 2000; Conrad et al., 2008; McCutcheon et al., 2011b). Also during withdrawal, dopaminergic signaling via volume transmission is reduced (i.e. hypodopaminergic tone), and downregulation of dopamine D_2_ receptors is observed, both of which contribute to reduced inhibitory feedback signals (Nutt et al., 2015; Volkow and Morales, 2015). These changes are linked to impulsivity and compulsive drug seeking (Lee et al., 2009; Moeller et al., 2018). The combination of reduced dopaminergic and glutamatergic signaling also contributes to aberrant plasticity during drug withdrawal (Huang et al., 2017). Gray-shaded boxes in the “withdrawal + rTMS” MSN dendrite represent proposed and speculative changes based on existing literature: 1. Upregulation of D_2_ receptors: rTMS over the PFC has been shown to alter extracellular glutamate and dopamine concentrations in the NAc, likely due to indirect activation of dopaminergic midbrain structures that project to the NAc. D_2_ receptor expression has been shown to be upregulated in the PFC following five daily sessions of rTMS in healthy mice (Etiévant et al., 2015). Chronic rTMS may therefore normalize the downregulation of D_2_ receptors in the NAc during withdrawal (D_2_ receptors, gray shading). 2. Insertion of GluA1-containing AMPA receptors: this has been observed within excitatory postsynapses of organotypic hippocampal slice cultures (Vlachos et al., 2012) and PFC of awake animals (Etiévant et al., 2015); however, it is not known whether this effect also occurs within NAc postsynapses and whether they also contain the GluA2 subunit (AMPA receptor, gray shading). Furthermore, it is not known whether the GluA2-lacking AMPA receptors that accumulate during withdrawal are affected by rTMS. mPFC, medial prefrontal cortex; DS, dorsal striatum; NAc, nucleus accumbens; VTA, ventral tegmental area; SN, substantia nigra; NMDA, *N*-methyl-D-aspartate; AMPA, α-amino-3-hydroxy-5-methyl-4-isoxazolepropionic acid; MSN, medium spiny neuron; rTMS, repetitive transcranial magnetic stimulation.

Stimulation of cortical regions that can alter activity and connectivity between regions is promising for alleviating the withdrawal symptoms in substance use disorders, particularly if it can be done non-invasively. In addition, because compulsive drug use has been associated with abnormal orbitofrontal- and mesolimbic-striatal circuits in subjects who are punishment resistant (i.e. even when faced with consequences, subjects continue to pursue the drug) (Hu et al., 2019), the possibility of using rTMS to stimulate hypoactive prefrontal cortical neurons, which can then modulate interconnected networks, is appealing (Diana et al., 2017; Madeo and Bonci, 2019; Song et al., 2019). An increasing number of studies have shown anticraving effects following rTMS treatment targeting the PFC (see Ma et al., 2019; Madeo and Bonci, 2019; Zhang et al., 2019), presumably through modulation of the efferent glutamatergic and afferent dopaminergic connections (Diana, 2011; Diana et al., 2017; Figure 1). Therefore, rTMS modulation of mesocorticolimbic pathways in people with substance use disorders may provide therapeutic effects.

Currently, the clinical studies that have utilized rTMS for treatment of addiction have varied protocols. This lack of consistency is common in rTMS research as there has not yet been a systematic approach to elucidate which parameters best achieve specific goals. Nonetheless, there is a general consensus on the target of stimulation: with the aim of modulating the mesocorticolimbic system, the majority of studies target the DLPFC, with only a few exceptions that stimulate the mPFC (Hanlon et al., 2015, 2017; Kearney-Ramos et al., 2018, 2019). In addition, most studies tend to stimulate only one side of the brain, usually the left, although a sham-controlled study comparing right- and left-side stimulations did not show a significant effect of laterality (Liu et al., 2017).

In 10-Hz stimulation, pulse numbers can range from 720 to 2,400 pulses per session, but 2,000 pulses per session are most common. The rationale is that excitatory stimulation to the PFC will increase the activity of glutamatergic corticostriatal efferents toward NAc and VTA; therefore, HF protocols are the most widespread and have been tested for potential anticraving effects in cocaine and methamphetamine use disorders. Excitatory stimulation generally uses 10- or 15-Hz protocols with an F-o8 coil, although recently there have been a few HF studies using H-coils (dTMS). Generally, 10-Hz stimulation uses a train duration of 5 s with an interstimulus interval (ISI) of either 15 or 10 s. There were only two exceptions for 10-Hz stimulations, one F-o8 study with 10-s train duration, 60-s ISI (Camprodon et al., 2007), and a dTMS study with 3-s train duration, 20-s ISI (Martinez et al., 2018). Similarly, 15-Hz stimulation addiction studies have trains of 60 pulses with 15-s ISIs with the exception of one dTMS study with trains of 36 pulses over 2 s with 20-s ISI (Rapinesi et al., 2016).

Protocols at 1-Hz deliver either 600 or 900 pulses, whereas protocols using cTBS usually deliver 3,600 pulses per session [one instance of 1,800 pulses/session (Hanlon et al., 2015)]. The total number of pulses also depends on the number of stimulation sessions. Within the field of addiction, the number of sessions varies across studies. Stimulation can be acute with a single active session, or chronic, with multiple sessions that range from 5 to 20 sessions applied either five or three times per week in clinical studies of addiction. Overall intensity of stimulation can range from 80 to 110% of rMT, with most studies using 100% rMT. Intensities at 100% rMT or below seem most suitable since several studies reported that intensities >100% rMT had poor tolerability and adverse effects among addiction patients (Su et al., 2017; Martinez et al., 2018).

Here we review the results of clinical studies that use rTMS as a treatment specifically for cocaine and methamphetamine abuse. A recent review of rTMS literature has suggested that the best predictor of rTMS-induced plasticity is pulse frequency (Wilson and St George, 2016); therefore, we have structured the studies by frequency of stimulation below.

### 5 Hz or Greater

The vast majority of addiction-related clinical rTMS studies use excitatory forms of rTMS in their studies. The goal is to try to increase the activity of the hypoactive frontal circuitry that is characteristic of the withdrawal stage of addiction, which is associated with a weakened executive control network and reduced dopaminergic transmission.

In clinical studies, HF-rTMS over the DLPFC has been shown to have anticraving effects (for an overview, see Ma et al., 2019). Most studies apply chronic stimulation (i.e. >4 stimulation sessions), once per day, but there are some studies that look at single session stimulation, with mixed results. For example, one study reported significantly lower craving scores (self-reported) for methamphetamine-dependent individuals after a single stimulation session for both left and right DLPFC at 10-Hz stimulation, with no change in the sham condition (Liu et al., 2017). Meanwhile a small sample of cocaine-dependent individuals had reduced craving in response to a single session of right DLPFC, but not left DLPFC, at 10-Hz stimulation (Camprodon et al., 2007), and another sham-controlled study found a single session of 10-Hz rTMS over left DLPFC induced no significant reduction in craving scores (Su et al., 2017).

Excitatory rTMS that is applied across multiple sessions (chronic) seems to have better and more reliable outcomes for substance abuse than single sessions. A recent meta-analysis that looked at single versus multiple sessions of neuromodulation across all addiction domains found that multiple sessions were more effective at reducing craving, with larger effect sizes compared to single sessions (Song et al., 2019). Recent systematic reviews have included several studies that demonstrate anticraving effects with chronic stimulation (Madeo and Bonci, 2019; Zhang et al., 2019). Moreover, in studies where there was no change in craving after the first session, there was a significant anticraving effect by the end of the treatment period (5 days of daily HF-rTMS) for active, but not sham, stimulation (Su et al., 2017). Furthermore, although there is often an underrepresentation of female patients in addiction studies, a recent study with 90 methamphetamine-dependent females showed that female subjects also respond well to chronic HF-rTMS, with significant anticraving effects compared to sham and waiting-list controls (Liu et al., 2019).

Although most clinical studies have applied 10-Hz stimulation protocols, there are also studies that have used 15-Hz stimulation protocols over the left DLPFC and shown significant decreases over time in both cocaine craving (Politi et al., 2008; Terraneo et al., 2016; Pettorruso et al., 2019) and cocaine use (measured by urine drug tests) (Terraneo et al., 2016; Pettorruso et al., 2019). However, so far, all 15-Hz studies have been open-label studies, without sham-controls. Although in one study, the rTMS group was compared with a control group treated with standard psychopharmacological treatments (Terraneo et al., 2016). Compared to the pharmacological controls, the rTMS group did have significantly lower craving scores and significantly more cocaine-free urine tests, supporting the therapeutic potential of rTMS (Terraneo et al., 2016).

In addition to anticraving effects, there have been reports that chronic HF-rTMS can improve withdrawal symptoms (Liang Y. et al., 2018; Pettorruso et al., 2019), anxiety and depression scores (Liang Y. et al., 2018; Pettorruso et al., 2019), sleep quality (Liang Y. et al., 2018; Lin et al., 2019), and several aspects of cognition (Su et al., 2017; Liang Q. et al., 2018). Therefore, chronic rTMS could be beneficial across several aspects of addiction, possibly due to changes in plasticity in the frontal cortex.

### Deep TMS

In addition to standard rTMS excitatory protocols, there have now been several HF-dTMS studies that use an H-coil, designed to deliver bilateral stimulation to deeper regions of the brain than is possible with an F-o8 coil and in a more diffuse manner. So far, three dTMS studies have been published looking at cocaine-dependent patients, and in all studies, a reduction in either intake or craving was reported for HF, multisession stimulation.

In an open-label study, craving was reduced compared to baseline midway through the treatment period, and this was maintained to the end of the treatment period (a total of 4 weeks) and 4 weeks after (Rapinesi et al., 2016). However, at the 4-week post-treatment follow-up, there was an increase in craving compared to the end of treatment, suggesting that maintenance sessions may be useful to keep cravings down (Rapinesi et al., 2016).

In a randomized controlled study using bilateral PFC stimulation and measurements of cocaine intake with hair samples, there was a significant reduction in intake over time regardless of stimulation group. However, there was no significant main effect of treatment and no interaction between time and treatment, suggesting that there was no difference between sham and rTMS intervention (Bolloni et al., 2016). However, the authors followed up with some exploratory *post hoc* testing looking at the effect of time on sham and rTMS data separately. Their *post hoc* findings show rTMS but not sham was associated with significant long-term reduction in cocaine intake at 2- and 3-month time points compared to baseline (Bolloni et al., 2016). Taking into account the low sample size and the risk of type 1 error from the exploratory *post hoc* testing, it is not clear whether dTMS is effective in reducing cocaine intake, but the exploratory results suggest that it is worth following up with a larger sample size.

Finally, a recently published randomized, sham-controlled study stimulated both the PFC and ACC (Martinez et al., 2018). They also introduced cocaine self-administration sessions, where participants were given the choice between a dose of smoked cocaine or a monetary reward in a progressive ratio task to measure the choice of cocaine when given an alternative reinforcer. Both HF (10 Hz) and LF (1 Hz) stimulation protocols were tested, but changes compared to sham were observed only for the HF group. There was no change in craving scores, but there was significant reduction in choice of cocaine after 13 sessions of HF-dTMS, 3 weeks in. In addition, the breakpoint of the progressive ratio was also lower for HF-dTMS in the third week (Martinez et al., 2018). This could suggest that after HF-rTMS participants were less willing to work for a reward, implying a drop in the incentive salience of the reward or a reduced motivational drive, both of which are responses underpinned by dopaminergic changes and associated with craving circuitry.

Overall, it is important to note that because of the different design of H-coils compared to other commonly used coils, and the relative paucity of dTMS addiction studies, it is still too early to conclude whether outcomes of the H-coil are markedly different compared to those of the F-o8 coils. Nonetheless, the promising early outcomes with dTMS raise the question of which aspects of the coil design and stimulation protocols are the most influential. Although H-coils are mainly associated with their depth of penetration, there are cone-shaped coils that can penetrate to similar depths (Deng et al., 2013). Double-cone coils (DCCs) have not been as widely used; however, they have been shown to be effective in treating disorders such as tinnitus (Vanneste et al., 2011; Vanneste and De Ridder, 2013; Kreuzer et al., 2015, 2018) and depression (Tastevin et al., 2019). In relation to addiction, there is limited research with an alcohol addiction case study showing marked reduction in craving with associated functional connectivity changes (De Ridder et al., 2011) and a recent study showing normalization of exteroception in cannabis users after posterior parietal cortex stimulation (Prashad et al., 2019). Although there are a few comparisons of DCC and F-o8 coil treatment (which have not shown any overall superiority of either coil) (Kreuzer et al., 2015; Tastevin et al., 2019), there are no comparisons between DCC and H-coil treatment. It has been mentioned that DCC stimulation may be less tolerable, and even painful, compared to H-coils due to the differences in field decay, but may achieve greater focality (Roth et al., 2002; Deng et al., 2013). These different coils could be directly compared in future trials. It may be that the capacity of the H-coil for bilateral stimulation and targeting of a large surface area with less intense stimulation (Deng et al., 2013) contributes to the effects of dTMS alternatively, or in addition to the depth of H-coil penetration.

### Intermittent TBS

So far, there have been no sham-controlled studies that have looked at the effectiveness of iTBS as a possible excitatory protocol to treat stimulant addiction. The shorter stimulation time and high efficacy compared to classic 10-Hz protocols have led to its growing popularity among rTMS therapies, particularly in major depressive disorder (Blumberger et al., 2018). There has, however, been a recent pilot study that compared two groups of treatment-seeking outpatients with cocaine use disorder that received either iTBS (3 min, 600 pulses/session, 80% active MT, *n* = 25) or 15 Hz (15 min, 2,400 pulses/session, 100% rMT, *n* = 22) over 4 weeks, with an accelerated protocol of twice-daily stimulations for the first week (Sanna et al., 2019). There was no significant difference in efficacy between the two protocols on measures of cocaine craving and consumption (Sanna et al., 2019), suggesting that iTBS may be as effective as 15 Hz in reducing cocaine consumption and craving. Intermittent TBS could therefore present advantages over 15 Hz because of the shorter stimulation time and lower intensity, which makes it more acceptable and tolerable for patients and more cost-effective for clinicians (Oberman et al., 2011). Although both treatment groups had large and significant reductions in consumption and craving after 25 days of treatment (Sanna et al., 2019), it is important to note that without a sham-control group a general effect of time or placebo response cannot be ruled out.

Interestingly, a small proof-of-concept, open-label study also found that an accelerated protocol of three times daily iTBS for 2 weeks significantly reduced cocaine intake and also nicotine, alcohol, and tetrahydrocannabinol intake in non-treatment-seeking cocaine-dependent individuals who had urine tests positive for cocaine (Steele et al., 2019). Usually, participants are required to test negative for drugs during treatment, so this study presents preliminary evidence that iTBS is effective and feasible as a treatment for active cocaine users.

### 1 Hz or Less

There are not many studies that have applied inhibitory protocols of rTMS to treat cocaine and methamphetamine addiction as addiction is primarily associated with hypoactivity of prefrontal cortices. However, a few studies have applied inhibitory protocols to methamphetamine and cocaine addicts, with mixed results.

Only two studies have looked at the application of 1-Hz stimulation in methamphetamine-dependent individuals (Li et al., 2013; Liu et al., 2017). The first study recruited non-treatment-seeking methamphetamine users in a sham-controlled crossover study and found that a single session of 1-Hz rTMS (900 pulses) over the left DLPFC increased cue-induced craving compared to the sham group, but not baseline craving (Li et al., 2013). In contrast, in a parallel, sham-controlled study, five sessions of 1-Hz stimulation (600 pulses/session) over either left or right DLPFC significantly reduced cue-induced craving compared to pretreatment baseline immediately after the first session and at the end of the final session (Liu et al., 2017). The very different results of these studies could in part be explained by the fact that the study showing an increase in craving (Li et al., 2013) had recruited current users, although not positive for methamphetamine on the days of experiments. In contrast, the study showing a reduction in craving consisted of participants who were all in rehabilitation, having stopped methamphetamine in the last 2 months (Liu et al., 2017). In support, animal studies show that α-amino-3-hydroxy-5-methyl-4-isoxazolepropionic acid (AMPA) receptor accumulation is different between stages of addiction (Scheyer et al., 2016), reviewed in the section “Glutamatergic Systems.”

### Continuous TBS

Similar to iTBS, cTBS is a short protocol, which can have greater effects on cortical inhibition than the classic 1-Hz inhibitory protocols (Huang et al., 2005). Below, we discuss a series of studies that apply acute cTBS over the mPFC in cocaine-dependent individuals, paired with functional magnetic resonance imaging (fMRI) and cue-reactivity tasks to look at changes in craving and brain activity. These are the only cocaine and methamphetamine addiction studies that use fMRI to investigate changes in brain activity and functional connectivity after rTMS. Their rationale is that cTBS, as an inhibitory protocol, may induce long-term depression (LTD)-like effects and dampen the activity of attentional and salience networks activated by drug-related cues (Hanlon et al., 2017).

Preliminary sham-controlled data from 11 chronic cocaine users after a session of cTBS (1,800 pulses/session) over the mPFC showed reduced fMRI activity in the insula, middle temporal gyrus, thalamus, and caudate regions compared to sham stimulation (Hanlon et al., 2015). However, there was no significant attenuation of craving compared to sham (Hanlon et al., 2015). In a larger, sham-controlled follow-up study that included chronic cocaine users, cTBS (3,600 pulses/session) over the left mPFC reduced activity compared to sham in the striatum, ACC, and parietal cortex (Hanlon et al., 2017). These regions can be linked to salience-processing (ACC) (Seeley et al., 2007), attention/executive control (parietal cortex) (Seeley et al., 2007), and craving (striatum) (Kober et al., 2010). The dampening of the salience network and reward processing by cTBS could be promising for reducing salience of drug-related stimuli and drug-cue craving. However, despite the changes in brain activity reported, there was no significant change in craving after cTBS compared to sham (Hanlon et al., 2017).

In a continuation of this line of investigation, a recent study added a cue-reactivity task before and after receiving real or sham cTBS (left mPFC, 3,600 pulses/session) to assess state-dependent effects of rTMS (Kearney-Ramos et al., 2018). In addition, during stimulation, participants were asked to think about and describe the last time they used cocaine, rather than simply being at rest. For cocaine users at baseline, drug-related cues elicited significantly higher functional connectivity between the mPFC and both striatal and salience-related regions compared to neutral cues (Kearney-Ramos et al., 2018). Following cTBS, the frontal connectivity for drug versus neutral cues was attenuated compared to sham, although there was no significant interaction for any region of interest, indicating a general effect across all regions (Kearney-Ramos et al., 2018).

Because there is considerable evidence for variability of rTMS effects/responsiveness across the population (Ridding and Ziemann, 2010), one study took a different approach and assessed whether baseline activity of striatum could be predictive of response to rTMS (Kearney-Ramos et al., 2019). Participants performed a similar task to the previous year’s study with cue recollection during cTBS stimulation over the mPFC (3,600 pulses/session) and a cue-reactivity task during fMRI, before and after cTBS (Kearney-Ramos et al., 2019). They found that baseline striatum activity during the cue-reactivity task predicted treatment response. High striatum activity during baseline cue-reactivity task resulted in reduced striatal activity after treatment, whereas low baseline striatum reactivity was associated with enhanced activity after treatment (Kearney-Ramos et al., 2019). The authors suggest that baseline striatal activity could act as a biomarker to identify positive rTMS responders, implying that state dependency arising from baseline neural activity can account for individual differences with rTMS (Kearney-Ramos et al., 2019).

Overall, there was no significant treatment-related change in general- or cue-induced craving for any of the cTBS studies; however, there were clear changes in functional connectivity, supporting the rationale for using rTMS to alter functional circuitry within the mesocorticolimbic pathways. As discussed previously, multiple stimulation sessions may be required before significant anticraving effects of rTMS can be detected. Accordingly, a clinical trial with multiple sessions using cTBS stimulation over the mPFC has been registered and is expected to be completed in 2020 (Hanlon, 2019, ClinicaTrials.gov identifier: NCT03238859), hopefully shedding light on the potential benefits of chronic cTBS for cocaine addiction.

## *R*TMS Effects Relevant to Treating Addiction – Linking Preclinical and Clinical Research

Among the many experimental protocols we describe above that aim to alleviate cocaine and methamphetamine use disorders, promising results from novel therapeutic regimes specifically relate to the potential of rTMS to induce anticraving effects (Diana et al., 2017; Madeo and Bonci, 2019). It is generally accepted that craving and relapse in individuals addicted to stimulant drugs such as cocaine and methamphetamine are associated with dysregulation of dopaminergic and glutamatergic systems (Diana, 2011; Diana et al., 2017; Madeo and Bonci, 2019). However, most of the clinical studies discussed above have similar overall designs and are not able to fully explore the mechanisms behind their therapeutic effects; therefore, basic research findings, in both healthy humans and animal models, offer another avenue to help understand the specific mechanisms underlying rTMS therapy.

Here, we review evidence that modification of glutamatergic and dopaminergic function may underlie the therapeutic effects of rTMS in individuals with cocaine and methamphetamine use disorders. We consider these therapeutic effects in the context of changes described in these circuits by experiments in laboratory animals (healthy animals and animal models of addiction) and in healthy humans. Our goal is to provide a mechanistic insight and highlight gaps in the literature that will ultimately facilitate translation and improvement of the current outcomes of rTMS therapy in addiction.

### Dopaminergic Systems

Dopamine is a critical neurotransmitter and neuromodulator for the induction and maintenance of neuroplasticity, a process related to learned behaviors (Jay, 2003; Wise, 2004; Kalivas and O’Brien, 2008). Convergence of excitatory and dopaminergic inputs appears necessary for the induction of long-term potentiation (LTP) (i.e. a Hebbian-type increase in synaptic strength) within the striatum. In particular, coactivation of D_1_-like receptors (Beninger and Miller, 1998; Smith-Roe and Kelley, 2000; Reynolds and Wickens, 2002) is crucial for reward-related instrumental learning (Wickens et al., 2007; Wickens and Arbuthnott, 2010). The repeated elevation of dopamine levels induced by stimulants such as cocaine and methamphetamine can surpass levels produced by biological stimuli, for which tolerance would normally occur. Such high levels of dopamine may therefore facilitate the abnormal learning or reinforcement of cues associated with the drug and thus initiate drug-seeking behavior (Kalivas and O’Brien, 2008). Repeated amphetamine exposure has been shown to accelerate habit formation (Nelson and Killcross, 2006), suggesting that the transition from voluntary, goal-directed responding to habitual drug use may be due to the recruitment of reward regulatory mechanisms from the ventral to dorsal striatum within the corticostriatal network, which then results in the expression of maladaptive incentive habits (Belin et al., 2009, 2013). In addition, cessation of drug use has been characterized by hypodopaminergic tone, particularly during the withdrawal phase, wherein a reduction of dopamine levels within the NAc is observed (Rossetti et al., 1992). Therefore, dopamine is critical for modulating synaptic plasticity within corticostriatal networks and may be relevant in the context of forming cue-induced drug craving (Wickens et al., 2007) and facilitating drug-seeking behavior by the weakening of executive functions (Arnsten and Li, 2005). Although it seems as though repeated elevation of dopamine levels drives network changes following exposure to drugs of addiction, such as the expression of aberrant synaptic plasticity and the hypodopaminergic tone within the mesolimbic system, dopamine may also be required during recovery (Nutt et al., 2015; Fattore and Diana, 2016).

The dopaminergic system appears susceptible to the effects of HF-rTMS as shown by changes in extracellular dopamine concentrations (microdialysis), or changes in protein concentration in the neuropil (brain homogenates). Although rTMS protocols vary widely between studies (Table 1), a consistent trend is an increase in dopamine within subcortical brain regions such as the striatum following rTMS. rTMS targeted to the frontal cortex has been shown to induce dopamine release in the rodent striatum (e.g., Keck et al., 2002; Kanno et al., 2004), and similarly, single-photon emission computed tomography imaging has shown a decrease in dopamine receptor binding after rTMS over the left DLPFC, suggesting an increase in extracellular dopamine in the caudate nucleus (Strafella et al., 2001) or general striatum (Pogarell et al., 2006, 2007). It was suggested that rTMS may have direct effects on striatal dopamine nerve terminals *via* corticostriatal projections, which is one pathway that can mediate subcortical dopamine release (Strafella et al., 2001). Other studies have also shown an increase in dopamine release in the NAc following stimulation of the motor cortex in humans (Strafella et al., 2003) and primates (Ohnishi et al., 2004). Although there has been no direct evidence of dopamine changes within the midbrain, only a limited number of studies have investigated this brain region (Ben-Shachar et al., 1997; Hausmann et al., 2002). Future studies that can more specifically probe changes within the mesocorticolimbic pathway would be valuable for understanding the effects of rTMS in addicted individuals.

Importantly, dopamine function is determined not only by the levels of dopamine, but also by synthesis and metabolism of the neurotransmitters and expression of its receptors and transporters. There is emerging evidence that HF-rTMS may affect these processes; for example, dopamine and its metabolite DOPAC have been shown to be increased in rat brain homogenates following 25-Hz stimulation (Ben-Shachar et al., 1997). A more recent study found that concentrations of DOPAC were altered in the striatum following stimulation at 10 Hz, although dopamine concentrations were not affected (Poh et al., 2019). Chronic stimulation has shown an increase in dopamine transporter mRNA that can last up to 10 days following the last stimulation session within the mouse cerebrum, as well as an increase in dopamine uptake, as measured in mouse synaptosomes (Ikeda et al., 2005). To our knowledge, there has been one study showing a change in dopamine receptor expression following rTMS. Five days of 15-Hz rTMS delivered to the frontal cortex in awake mice resulted in an upregulation of D_2_ receptor expression in the PFC (Etiévant et al., 2015). Therefore, rTMS may normalize the downregulation of D_2_ receptors that is observed in individuals with cocaine and methamphetamine use disorders. Taken together, these studies indicate that HF-rTMS has the capacity to alter dopamine release, uptake, and the activity of enzymes related to dopamine metabolism.

There is limited research looking at the effects of LF-rTMS on dopamine; however, a recent study looked at positron emission tomography scans of healthy volunteers following bilateral 1-Hz stimulation of the insular region using an H-coil (dTMS). They showed a decrease in dopamine neurotransmission in the substantia nigra, sensorimotor striatum, and associative striatum. Interestingly, there was no effect of 10-Hz stimulation on dopaminergic neurotransmission in the same study, yet these results suggest that it is possible to have an inhibitory effect on dopamine if the appropriate rTMS protocols are applied (Malik et al., 2018).

### Glutamatergic Systems

Although dopamine is the neurotransmitter most associated with addiction, glutamate is suggested to play a significant role in reinstatement of drug-seeking behavior after withdrawal (Wolf and Ferrario, 2010). Glutamatergic systems are best known for their key role in supporting synaptic plasticity processes such as LTP (strengthening of synapses) and LTD (weakening of synapses), which are integral in rTMS-induced neuroplasticity (Vlachos et al., 2012; Tang et al., 2015; Cirillo et al., 2017). In the case of cocaine-induced reinstatement of drug-seeking behavior, glutamate activity *via* the AMPA receptors in the NAc appears to be essential (Cornish and Kalivas, 2000; Conrad et al., 2008). For example, when AMPA/kainate receptor, but not *N*-methyl-D-aspartate (NMDA) receptor, activation is blocked in rats, there was no reinstatement of cocaine-seeking behavior in response to an injection of either AMPA or dopamine. Yet, when dopamine receptors were blocked, injection of AMPA still initiated drug-seeking behavior (Cornish and Kalivas, 2000).

Insertion and removal of AMPA receptors at the synapse are related to synapse strengthening (LTP) and weakening (LTD), respectively (Feldman, 2009; Kessels and Malinow, 2009). Subunit composition is also important as GluA2-lacking AMPA receptors are Ca^2+^-permeable and thus important for the induction of synaptic plasticity. In contrast, GluA2-containing AMPA receptors are Ca^2+^-impermeable, predominantly expressed in mature neurons, and their expression is associated with scaling down synaptic strength (for a review, see Liu and Zukin, 2007). Expression of LTP in the NAc especially during cocaine and methamphetamine withdrawal is associated with the accumulation of Ca^2+^-permeable AMPA receptors in the NAc, which results in an increased sensitivity of NAc neurons to excitatory inputs (Cornish and Kalivas, 2000; Conrad et al., 2008; Purgianto et al., 2013; Volkow and Morales, 2015; Scheyer et al., 2016), and is a requirement for cue-induced drug craving (Cornish and Kalivas, 2000; Conrad et al., 2008; McCutcheon et al., 2011b).

Interestingly, the group I metabotropic glutamate receptor (mGluR1) in the NAc appears to be involved in the development of the “incubation” period of cocaine or methamphetamine craving, which is defined as the progressive increase in cue-induced craving for the drug following withdrawal (Mameli et al., 2009; McCutcheon et al., 2011a; Scheyer et al., 2016). Activation of mGluR1 is able to reverse the accumulation of GluA2-lacking AMPA receptors in the NAc, which suggests that this receptor may be a potential target for addiction therapies to reduce cue-induced drug craving (McCutcheon et al., 2011a; Dravolina et al., 2017). Overall, these experiments suggest that glutamate initiates drug-seeking behavior in relapse, in contrast to dopamine, which is involved in the maintenance of drug-seeking motivation, and not an essential component behind AMPA-evoked craving.

Most studies investigating rTMS effects on glutamatergic circuits have investigated cortical and hippocampal structures. At high intensities, rTMS can evoke action potentials in neurons, and a single TMS pulse has been shown to induce a transient activation of voltage-gated Na^+^ channels (Banerjee et al., 2017; Li et al., 2017). Consequently, multiple HF pulses have been shown to induce LTP-like synaptic plasticity in the hippocampus and alter glutamate transporter gene and protein expression *via* miniature excitatory postsynaptic currents and alter dendritic spine sizes up to 6 and 3 h after magnetic stimulation, respectively, in CA1 pyramidal neurons located in the stratum radiatum (Vlachos et al., 2012). This strengthening of glutamatergic synapses requires activation of Ca^2+^-dependent NMDA receptors, L-type voltage-gated Ca^2+^ channels, and voltage-gated Na^+^ channels (Vlachos et al., 2012; Lenz et al., 2015). In addition, upregulation of the density and size of GluA1-containing AMPA receptors was observed within the stratum radiatum after stimulation (Vlachos et al., 2012; Lenz et al., 2015). However, it is not known whether these AMPA receptors also contain the GluA2 subunit. In another study, GluA1 receptor expression, but not GluA2 receptor expression, was upregulated in the PFC following 5 days of 15-Hz rTMS (Etiévant et al., 2015). At lower magnetic field intensities, alterations to neuronal excitability following rTMS within layer V cortical neurons have also been observed up to 20 min after stimulation, although the mechanisms are not known (Tang et al., 2016). Interestingly, an LF 1-Hz rTMS protocol, which is generally associated with inhibitory effects, delivered to Sprague–Dawley rats daily for 14 days (400 pulses per day) increased the excitability of hippocampal CA1 pyramidal neurons as shown by depolarized action potential thresholds (Tan et al., 2013). Therefore, it appears that rTMS may be able to alter intrinsic properties and excitatory synaptic connectivity of hippocampal and cortical neurons, as well as the expression of their neurotransmitter receptors. These findings may therefore be relevant to addiction research as animal models of addiction exhibit aberrant plasticity within the mesocorticolimbic pathway, resulting in dysfunctional neuroadaptations. For example, the hypoactive glutamatergic efferent projections from the mPFC contribute compulsive drug-seeking behaviors, but stimulation of these projections may reverse some of the maladaptive behaviors (Chen et al., 2013).

While receptors such as GluA and mGluR directly mediate neuronal response to glutamate, transporters also have an important modulatory impact on neurotransmission by regulating extracellular glutamate levels and thus controlling the availability of glutamate to bind to receptors. Accordingly, expression of glutamate transporters is a potential contributor to the changes in glutamatergic neurotransmission reported in addiction. For example, glial glutamate transporter I (GLT1) is downregulated following chronic cocaine self-administration, potentially increasing the amount of glutamate available to bind to receptors and increasing glutamatergic transmission (Kalivas and Volkow, 2011). Few studies have looked at glutamate transporter expression following rTMS, but recently a global gene expression study of the mouse cerebrum following 20 days of rTMS has shown upregulation of the glutamate transporter genes EAAT4, GLAST, and GLT1 and downregulation of EAAC1 24 h after the last stimulation session (Ikeda et al., 2019). However, 10 days after the last stimulation session, all of these glutamate transporter genes were upregulated (Ikeda et al., 2019). These findings are the first to demonstrate changes in glutamate transporter gene expression, and it will be interesting in future studies to isolate RNA from specific cortical regions to assess regional differences and impact on areas within the mesocorticolimbic system such as the NAc. Overall, these studies taken together with others showing regulation of vesicular glutamate transporter I (vGluT1) and GLT1 in the cerebellum following different TBS protocols (Mancic et al., 2016) suggest that glutamate transporters are likely to play an important role in mediating rTMS effects and are worth further investigation as therapeutic targets in addiction.

An increase in NAc glutamate and dopamine concentration has been observed following a single session of 2-Hz rTMS (Zangen and Hyodo, 2002), an effect that has been observed following electrical and optogenetics stimulation of excitatory neurons of the mPFC region in rodents (Taber et al., 1995; Kim et al., 2015; Quiroz et al., 2016). In addition, another study found that glutamate concentration was immediately reduced in the striatum following 10-Hz rTMS (Poh et al., 2019). Altered neurotransmitter concentrations within the striatal neuropil may reflect changes within intraneuronal sites and may not necessarily reflect changes in extracellular glutamate release following magnetic stimulation. In contrast, other studies have shown that glutamate levels were unaltered, although they were assessed in other brain regions (Keck et al., 2002; Seewoo et al., 2019). Despite the varied findings, it appears that glutamate release and concentration within the striatum (dorsal and NAc) are altered following rTMS; however, more research (e.g., electrophysiological recordings) is required to understand the effects of rTMS within this brain region.

Consistent with evidence that rTMS can alter glutamatergic neurotransmission, rTMS has been used therapeutically to target the dysfunctional glutamatergic system in aged mice (16–17 months old). Hippocampal CA1 pyramidal neurons of aged mice exhibit a reduced number of evoked action potentials from an injected stimulating current and increased hyperpolarization after an action potential compared to mature mice (9–10 months old), indicating reduced excitability (Potier et al., 1992; Randall et al., 2012; Wang et al., 2015). However, after 14 consecutive days of 25-Hz rTMS, the excitability of CA1 neurons in aged mice was restored to levels seen in mature mice, which suggests that rTMS can “rescue” hypoactive neurons in aged mice (Wang et al., 2015). This experiment suggests that anticraving effects reported in addicted populations following HF-rTMS to the PFC (see below) may be related to an rTMS-induced increase in excitability of hypoactive PFC glutamatergic neurons in addicted individuals. The hypothesis could be tested by applying HF-rTMS to the PFC of rats that exhibit compulsive cocaine self-administration, as their PFC neurons have been shown to exhibit reduced excitability, compared to rats that do not compulsively seek cocaine (Goldstein and Volkow, 2011; Chen et al., 2013; Madeo and Bonci, 2019).

### Animal Models of Cocaine and Methamphetamine Addiction and rTMS

Although at the moment there are only a few studies that have applied rTMS to animal models of addiction and have had promising results, only one has investigated the effects of rTMS following stimulation over the frontal cortex. Following abstinence in morphine-sensitized rats, dopamine levels within the NAc can be acutely altered by a single session of HF-rTMS (20 Hz, 300 pulses) over the left frontal cortex (Erhardt et al., 2004). Morphine-sensitized rats had a significant increase in dopamine, which was sustained for 120 min after stimulation compared to baseline. Non-sensitized control animals who also received rTMS also showed increase in dopamine levels at 30 min after stimulation; however, the morphine-sensitized rats had significantly higher dopamine release compared with the control rats (Erhardt et al., 2004). A caveat of this study was that morphine-sensitized animals did not exhibit lower dopamine levels within the NAc at baseline, even though this would be expected in an animal model of addiction (Nutt et al., 2015); however, the authors attribute this to the low dose of morphine used (Erhardt et al., 2004).

The only other studies of rTMS in an animal model of addiction that we are aware of investigated how rTMS affected the development of methamphetamine-induced conditioned place preference (CPP) and the reinstatement of CPP after extinction (Wu et al., 2018a, b). The stimulation site in one study was between bregma and lambda skull sutures (Wu et al., 2018a) and was not reported in the second study (Wu et al., 2018b). However, large size of the stimulating coils (circular coil: 5-cm outer diameter, 2.5-cm inner diameter) relative to the size of a rat still means that the whole brain (i.e. including the PFC) was likely stimulated (Rodger and Sherrard, 2015; Tang et al., 2015).

In the experiment testing the development of methamphetamine-induced CPP, rTMS, or sham stimulation was given prior to a methamphetamine injection and placement in a conditioning chamber (Wu et al., 2018b). After 4 days of conditioning, CPP was tested three times (2, 4, and 6 days after the end of the conditioning/treatment period). LF stimulation, but not HF stimulation, significantly inhibited methamphetamine-induced CPP (Wu et al., 2018b). In addition, the expression of GABA_B_ receptor subunit 1 (R1), but not subunit 2 (R2), in the dorsolateral striatum was significantly decreased in the methamphetamine + 1-Hz rTMS group compared to sham (Wu et al., 2018b). Interestingly, GABA_B_R1 in the dorsal striatum has been linked with rewarding memories of drugs (Jiao et al., 2016) and may be associated with the ability of LF-rTMS to inhibit drug-induced CPP. Furthermore, GABA systems are also modulated by rTMS (Lenz and Vlachos, 2016); however, more extensive review of the potential role of GABA in rTMS treatment of addiction is beyond the scope of this review.

The other experiment looked at the effect of HF-rTMS on methamphetamine relapse behavior (Wu et al., 2018a). After the extinction of CPP behavior, rats were given rTMS for either 1 or 3 days. Twenty-four hours after the final rTMS treatment, a reinstatement test was performed, with methamphetamine injected before placement into the testing chamber. The group that received 3 days of rTMS did not show reinstatement of CPP behavior in the reinstatement test, suggesting 3 days of HF-rTMS can inhibit relapse behavior (Wu et al., 2018a).

### Altered Plasticity in Addiction: Implications for rTMS Treatment Efficacy

As alluded to in the previous sections, the molecular changes involving glutamate and dopamine function that result from addiction alter cortical plasticity of addicted individuals in a way that impacts rTMS effects (Shen et al., 2016; Huang et al., 2017). In a methamphetamine self-administration rat model of methamphetamine addiction, corticostriatal plasticity could not be induced after an electrical stimulation protocol in the addicted model, but was normal in saline-administering control rats, as measured by electrical recordings from rat brain slices (Huang et al., 2017). The methamphetamine self-administering rats also demonstrated a deficit in motor learning for a rotarod task compared to control rats (Huang et al., 2017). The impaired plasticity was associated with altered cortical–striatal synapse functioning. Protein analysis of AMPA and NMDA receptor subunit composition in comparison to control rats suggested that the reduced plasticity of methamphetamine-administering rats could be linked to insertion of calcium-impermeable glutamate NMDA receptor subunits in the dorsal striatum and motor cortex (Huang et al., 2017).

Although it is not possible in humans to measure corticostriatal plasticity directly, there is evidence for reduced plasticity in the motor cortex in addiction: methamphetamine-addicted individuals showed a lack of MEP potentiation and MEP depression after a single session of HF-rTMS and cTBS, respectively, when compared to a healthy control group (Huang et al., 2017). Methamphetamine-addicted individuals also performed worse on a motor learning task compared to healthy controls (Huang et al., 2017). When task performance data from all participants were matched with their amount of plasticity induction after HF-rTMS, there was a significant positive correlation, further suggesting the link between reduced plasticity and poor learning behavior.

Therefore, it is important to keep in mind that addicted individuals may have a reduced susceptibility to plasticity induced by rTMS, due to alterations in dopaminergic and glutamatergic systems, and this could be a barrier to rTMS therapy. Nonetheless, there are indications that this reduced susceptibility may be overcome; for example, facilitating dopamine signaling with a dose of L-DOPA during early alcohol withdrawal in rats restored the blunted plasticity and improved limbic memory disruption (Cannizzaro et al., 2019). It would be interesting to explore whether a similar boost in dopaminergic signaling, whether with L-DOPA or a dopaminergic receptor agonist, could be combined with rTMS to improve or hasten therapeutic effects by improving the cortical–striatal plasticity of addicted individuals.

Overall, despite their limited number, the studies in animal addiction models provide evidence supporting an influence of rTMS on different aspects of addiction. HF rTMS over the frontal cortex increases dopamine release in the NAc and offers evidence that the effects of rTMS may differ in drug-sensitized models compared to control or healthy models, highlighting the need for rTMS studies that specifically investigate a drug-dependent model (Erhardt et al., 2004). HF rTMS can inhibit relapse behavior (Wu et al., 2018a). Furthermore, LF-rTMS appears to prevent the formation of drug-induced rewarding memory by downregulating GABA_B_R1 (Wu et al., 2018b).

## Future Directions and Outstanding Issues

Here we have reviewed only two systems (dopaminergic and glutamatergic) of a complex network, focusing mainly on corticostriatal connections. Inputs from other regions such as the amygdala and hippocampus are also involved, as well as inhibitory systems (GABA). However, we hope that summarizing and integrating the current evidence from experimental and clinical research in this narrow focus will help lead research in a direction that could improve outcomes of rTMS therapy for cocaine and methamphetamine use disorders.

### Clinical Studies

#### Need for Consistency and Scientific Rigor

Current drawbacks of clinical studies, which have also been pointed out by recent reviews, include the lack of follow-ups after treatment and the lack of sham-controls in some studies (Ma et al., 2019; Madeo and Bonci, 2019; Zhang et al., 2019). Clinical studies should include follow-up measurements, sham-controls, and greater consistency of stimulation parameters between studies. This would help improve understanding of the temporal effects of rTMS on addiction and facilitate comparisons between studies. We also need a systematic approach to investigate the effects of stimulation parameters. This could allow us to identify which parameters reliably induce long-term changes in target pathways. Having an idea of the most effective parameters regarding dosage (i.e. number of pulses), intensity, and number of sessions (e.g., accelerated protocols; Steele et al., 2019) will significantly improve the reproducibility and impact of therapeutic rTMS.

#### Better Outcome Measures for Insights Into Mechanisms

Many studies rely solely on subjective measures of craving, most of which are simple rating systems such as the visual analog scale. Craving is the primary surrogate indicator of treatment success (Singleton and Gorelick, 1998) and has noteworthy association with later drug use (Weiss et al., 2003). However, the evidence of an association between craving and instances of relapse or drug consumption can sometimes be conflicting (Miller and Gold, 1994; Weiss et al., 1995). Adding at least one extra measure to look at consumption (which can be measured with objective drug testing), anhedonia, or withdrawal symptoms, for example, could help expand the evidence of the treatment potential of rTMS. Because addiction is a disorder that has several systems and pathways involved, there are multiple possible avenues through which rTMS could induce beneficial change. A range of outcome measures would help establish whether rTMS can treat different aspects of addiction and increase the opportunities to link future animal models of rTMS addiction therapy with the most relevant clinical outcomes and facets of addiction. Current evidence from cellular and animal models suggests that changes within the dopaminergic and glutamatergic systems are the primary mechanisms of rTMS-induced anticraving effects in humans. However, there is still a paucity of research that specifically investigates these rTMS-induced molecular and circuitry changes in the mesocorticolimbic system, particularly in an addicted model. As such, it is our opinion that there are multiple avenues of research involving rTMS and addiction that have rich, as-yet untapped potential, especially with regard to animal models of rTMS. Below, we identify some possible research questions that would be both interesting and beneficial to the field.

#### Animal Models of Addiction

Animal models of addiction occupy a key position in a translational pipeline because they allow exploration and optimization of rTMS parameters in a uniform and readily available addicted population. The few studies investigating rTMS in animal models of addiction show interesting and promising results (Erhardt et al., 2004; Wu et al., 2018a, b) and hint at further potential: for example, animal models could be used to explore the effects of rTMS on drug-sensitized dopaminergic systems based on the differences in accumbal dopamine after rTMS in morphine-sensitized versus non-sensitized rats (Erhardt et al., 2004). In addition, it would be interesting to investigate the effects of chronic rTMS on dopamine levels following cocaine abstinence. Other experiments that may provide insight into therapeutic mechanisms of rTMS, and how these can be optimized, include the characterization of receptor expression (e.g., GluA2-containing and -lacking AMPA receptors, D_1_–D_5_ receptors) and measures of dopaminergic tone in addicted subjects with or without rTMS intervention.

The relevance of animal studies in understanding rTMS effects in humans has recently been highlighted by neuroimaging studies showing that rTMS can induce similar changes in functional connectivity in rats and in humans (Cocchi et al., 2016; Seewoo et al., 2018, 2019). More specifically, chronic rTMS in healthy rats was associated with changes to addiction-related networks such as the cortical–striatal–thalamic and basal-ganglia networks, with chronic HF-rTMS potentiating interoceptive/default mode network connectivity and attenuating connectivity in the salience network (Seewoo et al., 2019). Surprisingly, there have been no equivalent studies describing the effects of chronic rTMS on functional connectivity in addicted rodents or human populations. However, acute studies following cTBS in humans have shown some promising changes in network activity and state-dependent effects that could be used as biomarkers for predicting the suitability of rTMS therapy for drug-dependent individuals (Hanlon et al., 2015, 2017; Kearney-Ramos et al., 2018, 2019). Designing experiments that can be run in parallel in both clinical populations and animal models and linked through matching MRI imaging data would be of great benefit to the field.

## Summary

A number of recent studies have shown promising effects of rTMS in treating cocaine and methamphetamine addiction by reducing craving, especially after chronic stimulation, and in some cases reducing consumption and withdrawal symptoms. These effects have been further confirmed by several meta-analyses reporting a treatment effect of rTMS over the PFC. Although the PFC to NAc glutamatergic pathway has been shown to be critical for the development of compulsive drug-seeking behaviors, effects of rTMS on the activity and aberrant plasticity present within this pathway have never been investigated. Despite these current limitations, mechanisms from the field of addiction and studies that have looked at the acute effects of rTMS on the dopaminergic and glutamatergic systems have given us an idea of some of the mechanisms that may underlie the therapeutic effects of rTMS in addiction. Moving forward, it is now imperative to take advantage of the well-defined animal models of substance use disorders to test whether rTMS can counteract the mechanisms that underlie addiction, informing both researchers and clinicians to improve outcomes of rTMS therapy in addiction.

## Author Contributions

EP and JM conceived and wrote the manuscript. JR edited the manuscript. All authors contributed to the manuscript revision, and read and approved the submitted version of the manuscript.

## Conflict of Interest

The authors declare that the research was conducted in the absence of any commercial or financial relationships that could be construed as a potential conflict of interest.
